# Implementation of deep learning in liver pathology optimizes diagnosis of benign lesions and adenocarcinoma metastasis

**DOI:** 10.1002/ctm2.1299

**Published:** 2023-07-06

**Authors:** Mark Kriegsmann, Katharina Kriegsmann, Georg Steinbuss, Christiane Zgorzelski, Thomas Albrecht, Stefan Heinrich, Stefan Farkas, Wilfried Roth, Hien Dang, Anne Hausen, Matthias M. Gaida

**Affiliations:** ^1^ Institute of Pathology Heidelberg University Heidelberg Germany; ^2^ Pathology Wiesbaden Wiesbaden Germany; ^3^ Department of Hematology Oncology and Rheumatology Heidelberg University Heidelberg Germany; ^4^ Laborarztpraxis Rhein‐Main MVZ GbR Frankfurt am Main Frankfurt Germany; ^5^ Department of Surgery JGU‐Mainz University Medical Center Mainz Mainz Germany; ^6^ Department of Surgery St. Josefs‐ Hospital Wiesbaden Germany; ^7^ Institute of Pathology JGU‐Mainz University Medical Center Mainz Mainz Germany; ^8^ Department of Surgery Department of Surgical Research Thomas Jefferson University Philadelphia Pennsylvania USA; ^9^ TRON JGU‐Mainz Translational Oncology at the University Medical Center Mainz Germany; ^10^ Research Center for Immunotherapy JGU‐Mainz University Medical Center Mainz Mainz Germany

**Keywords:** artificial intelligence, deep learning, liver metastasis, liver pathology, personalized medicine

## Abstract

**Introduction:**

Differentiation of histologically similar structures in the liver, including anatomical structures, benign bile duct lesions, or common types of liver metastases, can be challenging with conventional histological tissue sections alone. Accurate histopathological classification is paramount for the diagnosis and adequate treatment of the disease. Deep learning algorithms have been proposed for objective and consistent assessment of digital histopathological images.

**Materials and methods:**

In the present study, we trained and evaluated deep learning algorithms based on the EfficientNetV2 and ResNetRS architectures to discriminate between different histopathological classes. For the required dataset, specialized surgical pathologists annotated seven different histological classes, including different non‐neoplastic anatomical structures, benign bile duct lesions, and liver metastases from colorectal and pancreatic adenocarcinoma in a large patient cohort. Annotation resulted in a total of 204.159 image patches, followed by discrimination analysis using our deep learning models. Model performance was evaluated on validation and test data using confusion matrices.

**Results:**

Evaluation of the test set based on tiles and cases revealed overall highly satisfactory prediction capability of our algorithm for the different histological classes, resulting in a tile accuracy of 89% (38 413/43 059) and case accuracy of 94% (198/211). Importantly, the separation of metastasis versus benign lesions was certainly confident on case level, confirming the classification model performed with high diagnostic accuracy. Moreover, the whole curated raw data set is made publically available.

**Conclusions:**

Deep learning is a promising approach in surgical liver pathology supporting decision making in personalized medicine.

## Graphical abstract

Artificial intelligence algorithms allow the separation of anatomical, benign, and malignant structures in histological slides of the liver.

Digital image analysis on case level revealed overall highly satisfactory prediction capability of our algorithm for the different histological classes, resulting in a tile accuracy of 89% (38413/43059) and case accuracy of 94% (198/211).

A large curated data set of liver histology is provided for further optimization and research.

Our algorithm can be applied in surgical liver pathology supporting decision making in establishing the diagnosis.

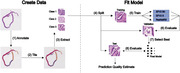

## 1 INTRODUCTION

Diagnosis of benign lesions, primary carcinoma, or metastases in surgical pathology relies heavily on histological evaluation using conventional methods, including hematoxylin and eosin stained and additional immunohistochemistry sections. Although these procedures are well established and used innumerably, in some cases an exact diagnosis is not possible. This often results from the fact that benign and malignant structures have similar histological appearance. Additionally, it can be challenging to discriminate between benign and malignant formations, which can arise side‐by‐side.^1^ Various structures within the liver may show a similar histological picture by formation of glandular, ductal, or tubular structures. In non‐neoplastic liver tissue, these are anatomical biliary ducts. Besides the preformed anatomical structures, benign gland‐like or ductal lesions in the liver can resemble bile duct adenoma, peribiliary hamartoma; all of which need to be confidently differentiated as non‐malignant from malignant liver lesions. Moreover, the exact histological differentiation is rather complex for biopsy specimens, since the sample size is small and can include both benign and/or malignant structures.

Once specimens are categorized as malignant, further histological subtyping is required for diagnostic purposes. In general, malignant gland‐like or ductal structures in the liver are adenocarcinoma metastases, the most common malignant neoplasms in the liver.^2^, ^3^ Notably, more than 5% of all cancer patients show synchronous liver metastasis, which underlines the high medical need of the correct diagnosis.^4^ However, the determination of the origin of adenocarcinoma metastases can be difficult for pathologists, since adenocarcinoma of the colo‐rectum or the pancreas shows similar heterogeneous growth patterns. They frequently present with glandular, ductular, or tubular patterns, but also cribriformic, papillary, solid, or even single‐cellular pattern can occur; all intermixed with surrounding desmoplastic stroma or necrotic areas. More often, various growth patterns are dispersed within the tumour.^5^ For the exact diagnosis, immunohistochemistry staining is often helpful.^6^ However, the majority of cases are diagnosed by biopsy and not up‐front resection, and thus, the amount of tissue for immunohistochemistry with multiple markers or molecular testing (e.g. mutation analysis, microsatellite status) is limited. As individualized and personalized medicine becomes a part of clinical routine, tissue‐saving diagnostic methods will be necessary to avoid multiple biopsies and ensure time sensitive therapy. In particular, unknown primary cancers are frequently diagnosed as liver metastasis using biopsy material with very little tumour content^6^

Besides the mentioned diagnostic aspects, an often unattended infrastructural point should be taken into account: the worldwide decreasing number of pathologists with an increasing number of (tumour) cases. Therefore, new resource‐saving methods with high diagnostic accuracy are desirable to support clinical routine diagnostics.^7^, ^8^ Most recently, digitalization was successfully implemented in surgical pathology for morphological‐based tissue slide analysis in routine diagnostic and research.^9^ Previously, it has been shown that deep learning algorithms can be used to classify scanned histopathological tissue sections, including the identification and differentiation of pancreatic anatomical structures, pancreatic intraepithelial neoplasms, and pancreatic cancer with high accuracy.^10^, ^11^, ^12^, ^13^, ^14^, ^15^ Deep neural networks, in particular convolutional neuronal networks (CNNs) are widely used algorithms for image classification.^16^ CNNs essentially consist of an input layer, multiple hidden layers for operations such as convolutions or pooling among others, and usually an output layer. For more detailed information on CNNs see Alzubaidi et al.^17^

To address these clinically highly relevant issues, including differentiation between benign and malignant liver lesions, histological subtyping, small amounts of tissue, and accelerating infrastructural obstacles, we performed the present study. Here, we train a CNN‐based deep learning network, which can accurately differentiate between liver metastasis benign lesions as well as identify and differentiate non‐neoplastic anatomical liver structures such as bile ducts and hepatocytes on scanned histopathological slides. Our network can be implemented in surgical pathology diagnostics to detect the most frequent types of adenocarcinoma metastasis in the liver with high accuracy and discrimination from benign structures. The trained model should provide more diagnostic confidence, uses small tissue samples, avoids multiple biopsies, assures diagnostic quality, and should help with the shrinking pathology resources. In addition, the provided huge raw dataset can be extended by pathologists and researchers to refine classification algorithms.

## 2 METHODS

**FIGURE 1:**
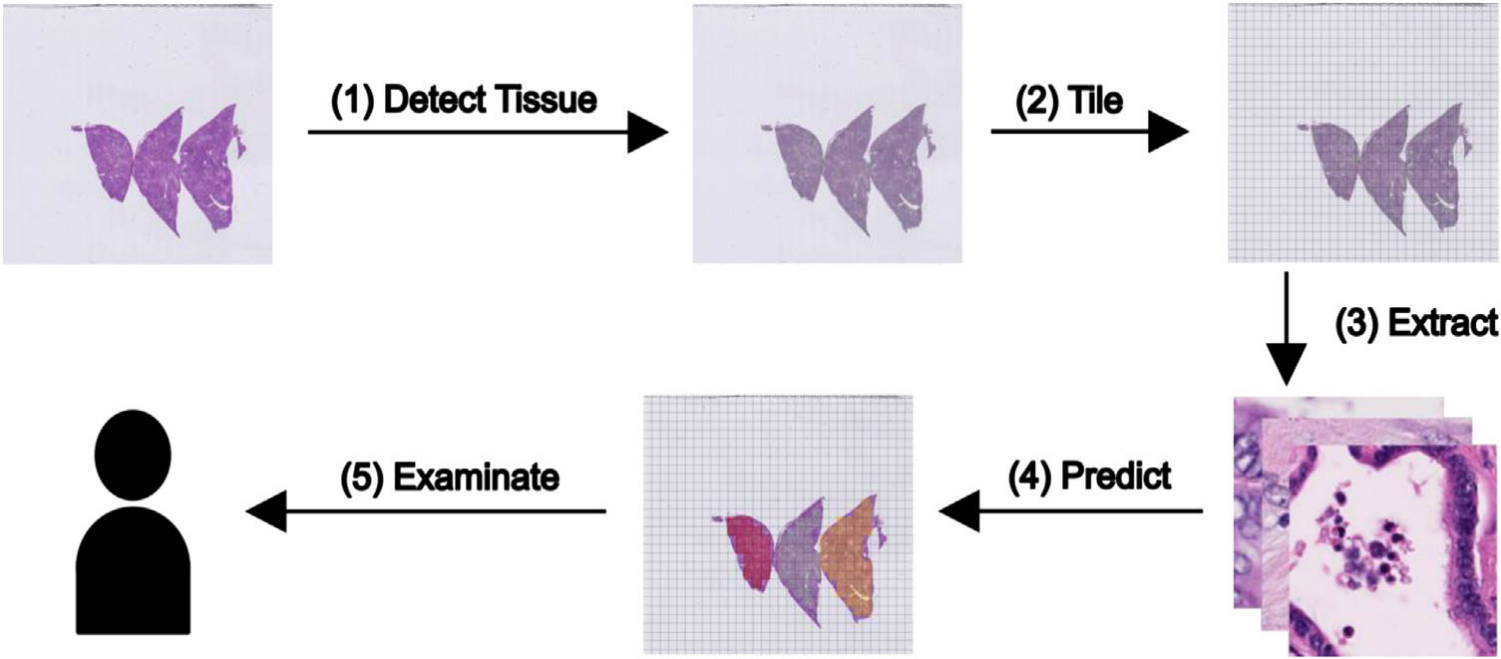
Workflow overview. The trained model can be used assist pathologist in their diagnosis by discriminating important classes within a given whole slide. In this study, we created data set of image tiles annotated by experienced pathologists and fitted a model to respective data.

Figure 1 displays a schematic overview of the anticipated clinical implementation of the proposed algorithms.

### 2.1 Patient data

Whole Hematoxilin and Eosin stained slides from patients with liver metastases of colorectal adenocarcinoma (*n* = 103), liver metastases of pancreatic adenocarcinoma (*n* = 101), bile duct adenoma and peribiliary hamartoma (*n* = 53) were extracted from the archive of the Institute of Pathology, University Hospital Mainz. Tissue samples were extracted from either resection specimen, needle biopsies, or intraoperative frozen sections. All diagnoses were made according to the World Health Organization Classification of Tumours of the Gastrointestinal Tract by board certified surgical pathologists, all with specialization in surgical liver pathology. All slides with representative tumour regions were scanned using an automated slide scanner (Aperio AT2, Leica Biosystems, Nussloch, Germany) with 400× magnification, as previously described. The correctness of annotated areas was cross‐validated by 4 independent surgical pathologists (A.H. and M.M.G. University Medical Center Mainz) and (T.A. and M.K. University Hospital Heidelberg). In all samples, a consensus was obtained.^18^ Image data were anonymized and are provided along with this manuscript (Link: https://doi.org/10.11588/data/YAZWJW).

The analysis was approved by the local ethics committee of Heidelberg University (#870/21) and of the University Hospital Mainz (approval 2019−14390; Ärztekammer Rhineland‐Palatinate).

### 2.2 Image data

**FIGURE 2:**
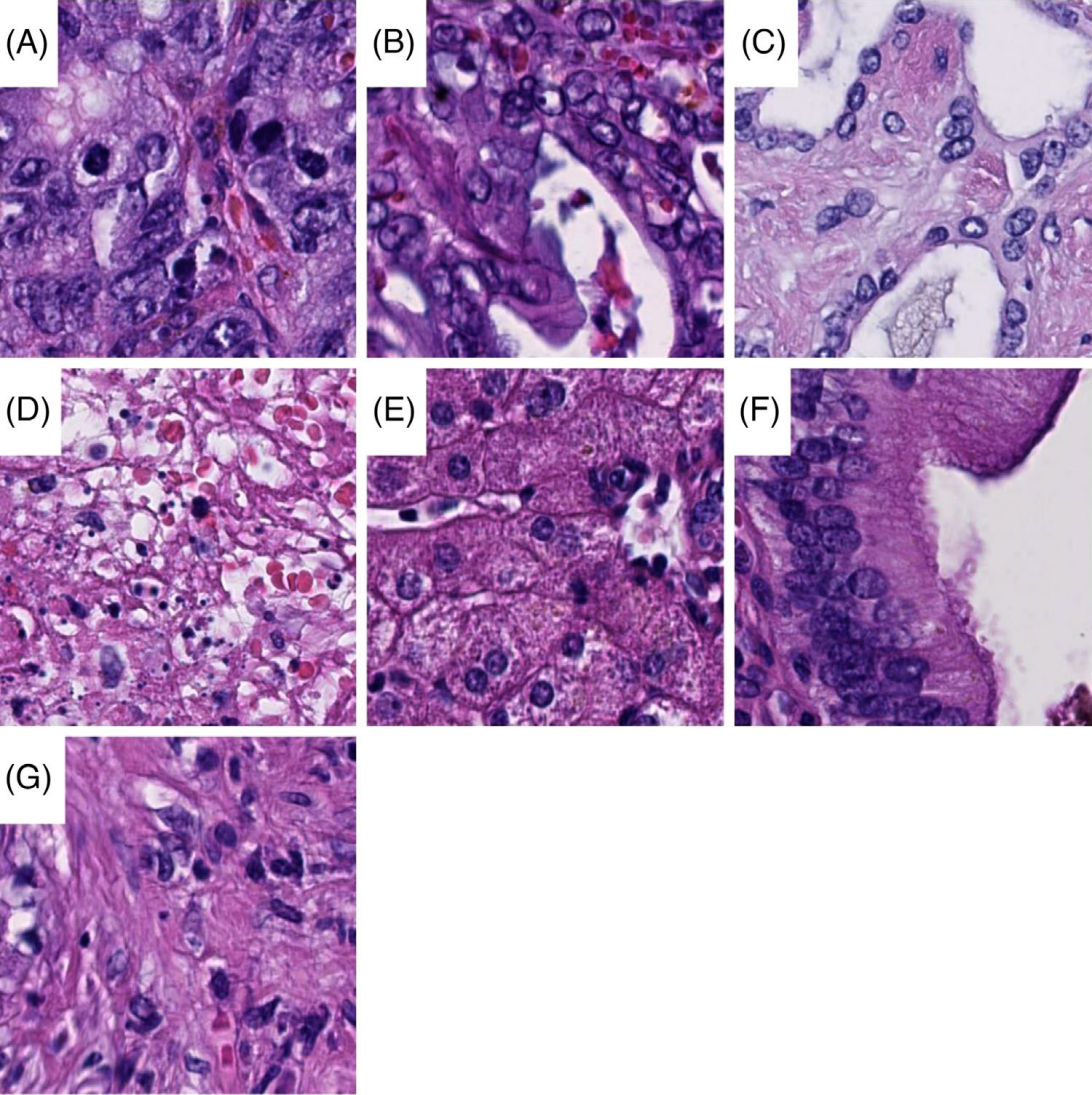
Examples of image tiles. Image tiles 100 × 100 μm in size from colorectal adenocarinoma (A), pancreatic adenocarcinoma (B), benign bile duct lesions (C), necrosis (D), hepatic cells (E), bile duct (F) and connective tissue (G) are displayed.

Scanned histopathological slides were imported into QuPath^19^ (v.0.1.2, University of Edinburgh, Edinburgh, UK) and annotated for the following 7 categories: liver metastases of colorectal adenocarcinoma, liver metastases of pancreatic adenocarcinoma, bile duct adenoma and peribiliary hamartoma, necrosis, non‐neoplastic hepatic tissue, bile ducts, and connective tissue. Image patches 100 × 100 μm (∼395 × 395 px) in size were generated in QuPath, extracted on the local hard drive and subsequently reviewed. Blurry images were deleted. The number of image patches per class is highlighted in Table 1. Representative image patches are displayed in Figure 2.

**TABLE 1:** Number of image patches and patients in the training, validation and test set.

Class	Training, *n* (%)		Validation, *n* (%)		Test, *n* (%)	
	By patches	By patient	By patches	By patient	By patches	By patient
Colorectal adenocarcinoma	23235 (60)	63 (61)	9140 (23)	21 (20)	6568 (17)	19 (18)
Pancreatic adenocarcinoma	13030 (57)	61 (60)	4395 (19)	20 (20)	5950 (25)	20 (20)
Bile duct adenoma and peribiliary hamartoma	4353 (63)	31 (58)	952 (14)	11 (21)	1627 (23)	11 (21)
Necrosis	11291 (52)	76 (59)	5074 (23)	26 (20)	5513 (25)	26 (20)
Hepatic tissue	40118 (57)	128 (58)	16225 (23)	46 (21)	14537 (21)	47 (21)
Bile ducts	4759 (61)	99 (60)	1539 (20)	35 (21)	1511 (19)	30 (18)
Connective tissue	19942 (58)	116 (62)	7047 (21)	37 (20)	7353 (21)	35 (19)

### 2.3 Splitting of datasets into training, validation and test set

Images from patients were separated into a training, a validation, and a test set with a target to achieve a distribution of 60% (training), 20% (validation) and 20% (test), respectively. All image patches from one patient were used in only one of the respective sets. Since a single patient may provide images for multiple classes, a naive random allocation of all patients to the three sets might not result in all classes being represented well in all three sets. Thus, we performed an iterative stratified sampling, described as follows. First we counted for each class the number of patients that provided images for the respective class. Then we iterated from the class with the lowest count to the class with the highest count. In each iteration, we randomly assigned patients to our sets that provide images for the respective class and have not already been assigned in a previous iteration to one of our three sets. The resulting sets were not changed during the analyses. The splits by image patches and patients are displayed in Table 1.

### 2.4 Hardware and software

For training we used a Graphics Processing Unit (GPU) instance from the bwForCluster Helix (https://wiki.bwhpc.de/e/Helix) service from Heidelberg University with a single GPU, 8 processors and 16 GB RAM. Further we used the Scientific Data Storage service from Heidelberg University. Training was performed using a singularity container image based on the TensorFlow Docker container image.

### 2.5 Training and validation of different models

Each model was based on the EfficientNetV2 or the ResNetRS architecture.^20^, ^21^ Each trained configuration makes use of random augmentation via the imgaug python module, uses a batch size of 128, the AMSGrad optimizer (a variant of the Adam optimizer^22^ with β1 = .9, β2 = .999 and ϵ = 1.0∗10^−7^) and during training the data are sampled such that there is no class imbalance and patients are not over represented. In particular within each epoch, we sample with replacement from all training tiles as follows:

1. We sample uniformly (each class is selected with equal probability) from the available classes.
2. Within each class, we sample the patients that provide tiles for these classes uniformly.

This strategy ensures that no class is under‐ or overrepresented during training. While classes are balanced with this strategy, it might be that patients which provide tiles for many classes are shown to the network more often than patients which provide tiles for fewer classes. Each epoch consists of 911 steps ( = $\left\lfloor \frac{\mathrm{TrainingImages}}{128}\right\rfloor$). We always use random augmentation with *N* = 2 and *M* = 10.

For each model configuration, the learning rate and the used architecture is trained six different times to account for the randomness involved in training a model (e.g., the random weights initialization). We display Matthews correlation coefficient (MCC) (cf. ‘metrics.MatthewsCorrelationCoefficient’ in python package tensorflow_addons) and the macro average of the area under the receiver operating characteristics curve (AUC_MA) (cf. ‘keras.metrics.AUC’ in python package tensorflow) to indicate overall prediction accuracy. Each model was proposed with different input image sizes and we resized our tiles to the particular size a model required via the TensorFlow function tf.image.resize.

The baseline model (EfficientNetV2B0) was trained with a learning rate of .01 and .001, an input size of 260 × 260 px, a dropout of .2 and *M* = 10 for random augmentation.

Next, we scaled up, that is, input size, network depth and width. In particular, we used the S instead of the B0 architecture (EfficientNetV2S). These models were trained with learning rates .01, .001 and .0001 and an input size of 384 × 384 px.

Last, the ResNetRS50 architecture was trained with learning rates .01, .001 and .0001, each with an input size of 224 × 224 px.

We compared the different configurations (application and learning rates) by plotting the respective best epochs for each trained model. In particular, for each model we plotted the five best epochs according to the validation AUC_MA and the five best epochs according to MCC. Moreover, the training times were compared between the three architectures as a surrogate for the inference time.

To evaluate the performance of the models, confusion matrices for image tiles and patients were created and compared for validation and test data. Each confusion matrix shows the number of tiles in respective actual and predicted class, along with the percentage of actual tiles affected. That is, the percentages in a row add up to 100%. For case‐based results, majority prediction of all tiles of a specific class was calculated from that case. That is, a case is composed of all tiles of a specific class (by annotation of a pathologist) from a specific whole slide. We excluded the EfficientNetV2S architecture form this evaluation, since its performance was not significantly better compared to the other models, but training times were much higher.

To visualize the similarity of the different classes, the uniform manifold approximation and projection (UMAP) was plotted of the activation layer just before the top dense layer (after the last average pooling layer) for tiles and for cases. For cases, the median of tile based coordinates were used.^18^

All our codes are available at https://doi.org/10.11588/data/YAZWJW.

## 3 RESULTS

### 3.1 Training, optimization and validation of different models

The tissue sections were *lege artis* separated into a training cohort (60%), a validation cohort (20%), and a final test cohort (20%). This resulted in a total of 116.728 image tiles for training, 44.372 for validation and 43.059 for testing, out of in total 204.159 image patches (cf. Table 1 for detailed numbers). During training, the CNNs are optimized for the classification of the images in the training set. The validation set is used for comparing model performance, since the performance on the training set is not always reliable in terms of generalization to other images. A model that memorizes training images and respective classes will give a high training performance. However, with images different to the training images such models will fail to predict the correct class.

**FIGURE 3:**
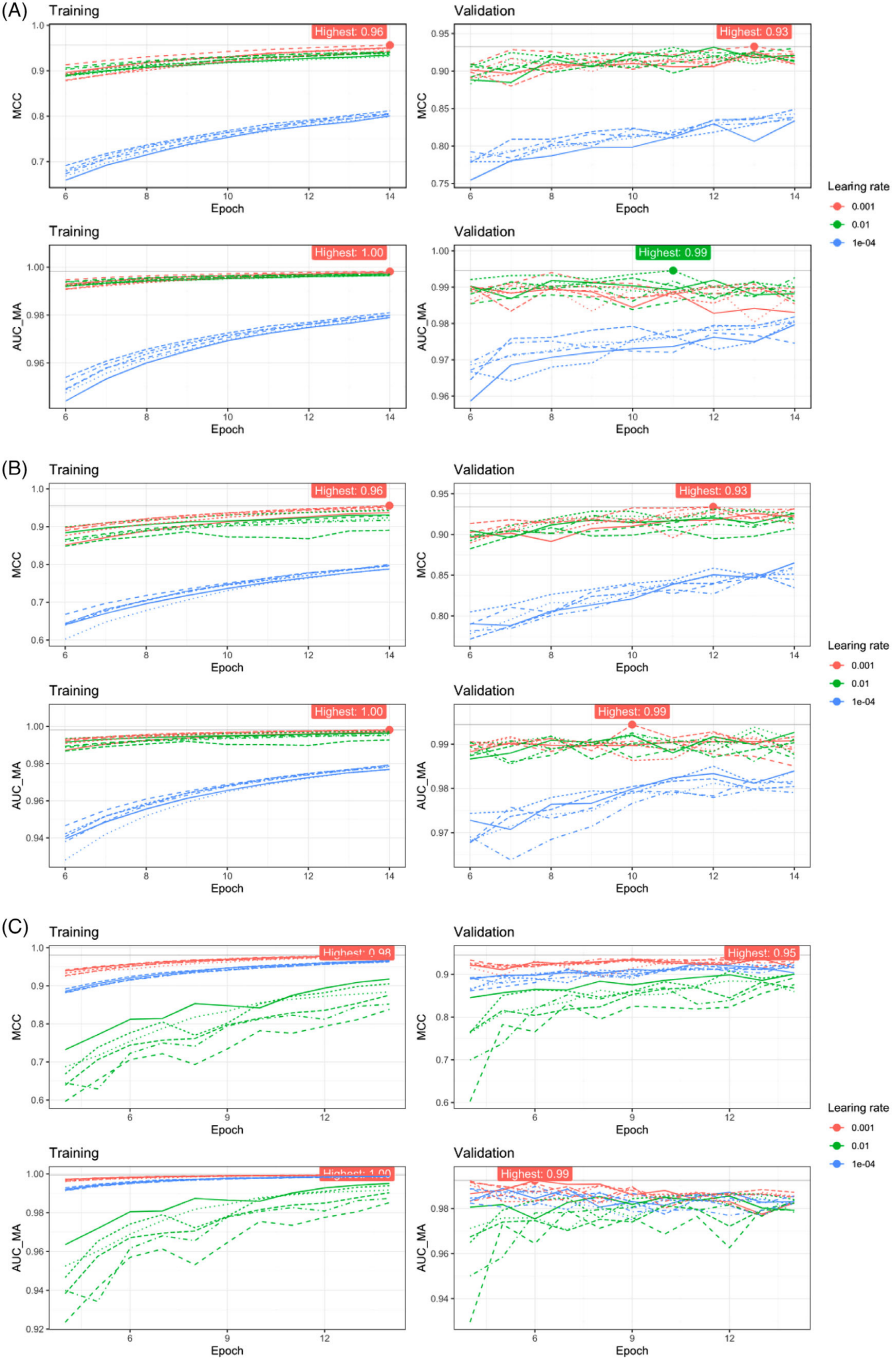
Models trained. With the EfficientNetV2B0 architecture (A), the EfficientNetV2S architecture (B) and the ResNetRS50 architecture (C) a learning rate of .001 showed a higher Matthews correlation coefficient (MCC) as compared to a learning rate of .01. Dashed and solid lines correspond to the six different trainings with the same parameters to account for randomness (e.g., the random weights initialization).

The learning rate is a crucial parameter for the optimization of models to the training data. Thus, we trained our models using different learning rates and compared the trained models using the validation data. Initial training of models with the EfficientNetV2B0 architecture indicated a learning rate of .1 being too large: respective models diverged (AUC ∼.5). Hence, this learning rate was not further used. Models trained with a learning rate .01 and .001 seem to perform best (AUC > .99), cf. Figure 3A.

In addition to the learning rate, the model architecture can affect the prediction quality. Hence, we also tested a larger model using the EfficientNetV2 architecture (EfficientNetV2S) and a model of a different architecture (ResNetRS50). Using the larger model (EfficientNetV2S), we could again observe that learning rates of .01 and .001 performed best (Figure 3B), but the larger models did not outperform the smaller models: both result in similar AUC and MCC on the validation data (Figure 3A and Figure 3B). With the ResNetRS50 architecture, a learning rate of .001 resulted in best performance (Figure 3C) and resulted in a similar AUC and MCC on the validation data as well.

**FIGURE 4:**
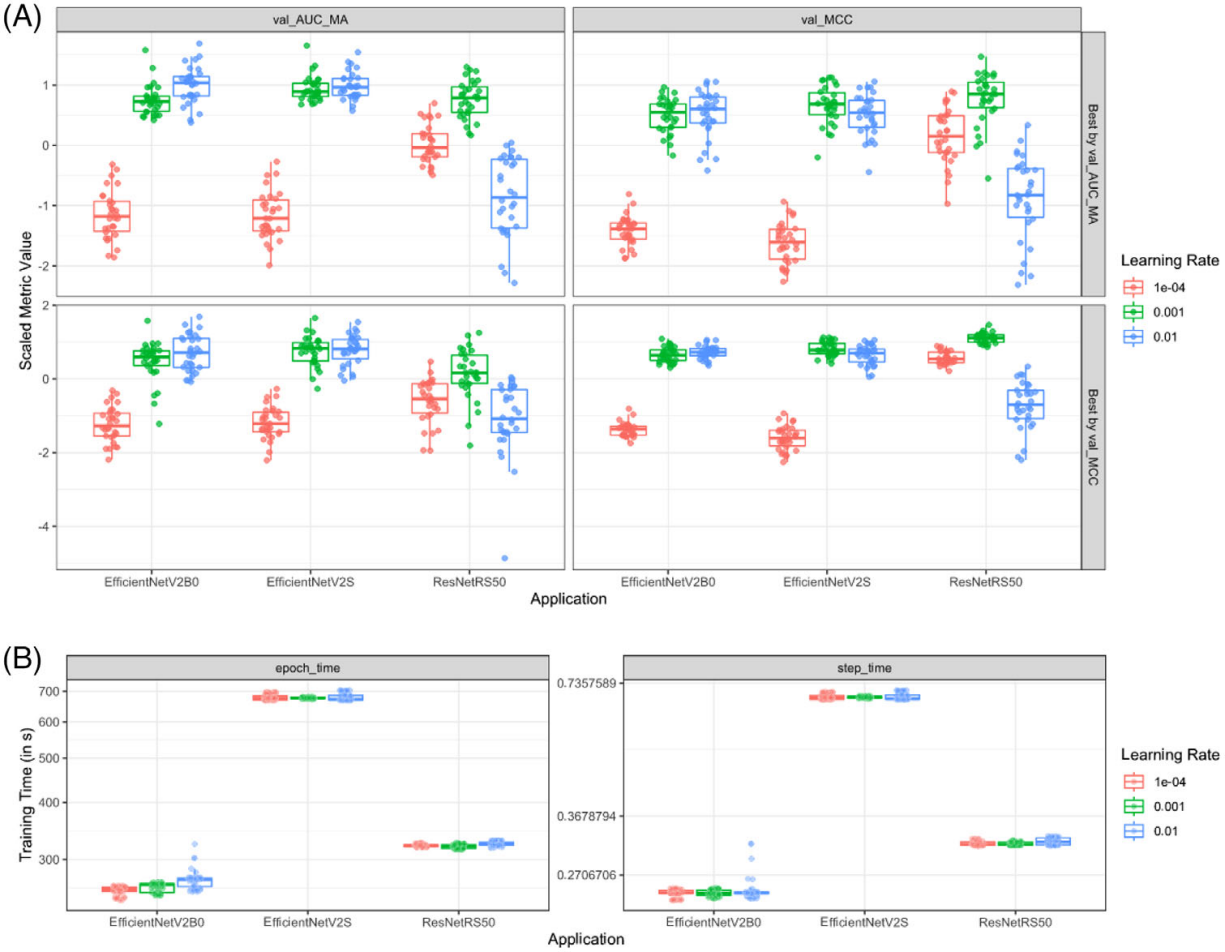
Comparison of the three model architectures. A learning rate of .001 performed well on all three architectures regarding Matthews correlation coefficient (MCC) and accuracy (ACC) (A). The training times of the EfficientNetV2B0 and the ResNetRS50 were similar and much lower as compared to the larger EfficientNetV2S architecture (B).

Figure 4A displays a comparison of all trained models. For each architecture and learning rate, we compared the validation AUC and MCC of the five best epochs (in terms of validation MCC) of respective models. Within each metric the values are scaled to facilitate a simpler comparison. Figure 3A shows that with the EfficientNetV2 architecture, a learning rate of either .01 or .001 performed well (Figure 3A and B), with ResNetRS50 models a learning rate of .001 is better (Figure 4A). Considering the MCC of the validation data, the ResNetRS50 outperformed the other models slightly, since the respective box is slightly higher. However, according to the AUC the EfficientNetV2 models perform slightly better.

The computational time needed for training and inference is an important aspect for the usefulness of our models: specifically, a higher inference time does impose significant costs associated with using the models routine diagnostic. In this regard, Figure 4B compares the recorded training times for each model. The ResNetRS50 models training speed is similarly to the training speed of the EfficientNetV2B0 models. Both models train about two times faster than the larger EfficientNetV2S architecture. This was expected, since both EfficientNetV2B0 and ResNetRS50 are very small models in terms of the number of weights (compared to EfficientNetV2S or other common ResNetRS models) and the ResNetRS models are competitive to EfficientNet in terms of speed and accuracy. Notably, we assume that the inference time will behave similar to the training times and as a result, we excluded the EfficientNetV2S models from further comparisons due to their much slower training speed but similar prediction quality.

The two best performing models for the two remaining architectures EfficientNetV2B0 and ResNetRS50 are summarized in Table 2.

**TABLE 2:** Best performing models based on MCC on validation data.

Model	Epoch (*n*)	MCC (val. data)	AUC_MA (val. data)	Learning rate	i (*n*)
EfficientNetV2B0	13	.93	.99	.001	4
ResNetRS50	13	.95	.99	.001	3

Abbreviations: AUC, accuracy; AUC_MA, Macro average of the area under the receiver operating characteristics curve; i; number of trained model (six models were trained with each change of parameters; MCC, Matthews correlation coefficient.

To select the final model, we investigated the validation data based on tiles and patients in more depth. Confusion matrices show good performance of both models, reflected as high values on the diagonal. Problems were noted mainly in the discrimination between liver metastases of colorectal adenocarcinoma and liver metastases of pancreatic adenocarcinoma (Figure S1A,B).

A UMAP of the activation layer just before the top dense layer is a visual tool to check the representation of tiles, which the network has learned and thus is another tool to compare model performance. The representations displayed in the UMAP are used by the network for the classifications. Ideally, all classes should be well separated in the UMAP. This indicates that the internal representation of images for the different classes is also very different within the network; and, hence, the network can distinguish them well. Respective UMAPs of the models from given in Table 2 show slightly less sprinkled class clusters with the EfficientNetV2B0 model (Figure S2A,B). However, the overall separation between classes for both models seems similar. The UMAP summarized to patient level show not much difference to the tile level (cf. Figure S2A vs. S2B).

Based on these performances, we selected the EfficientNetV2B0 model for evaluation of the test set.

### 3.2 Evaluation of the test set

Since the final model was selected via its performance on the validation data, respective prediction quality is not an honest measure of the prediction accuracy when using new images. Therefore, we performed the evaluation of the model on the test set.

Evaluation of the confusion matrices for the test set based on tiles and cases revealed overall highly satisfactory prediction capability of our algorithm for the different classes (tiles: accuracy = 89% [38413/43059]; cases: accuracy = 94% [198/211]).

**FIGURE 5:**
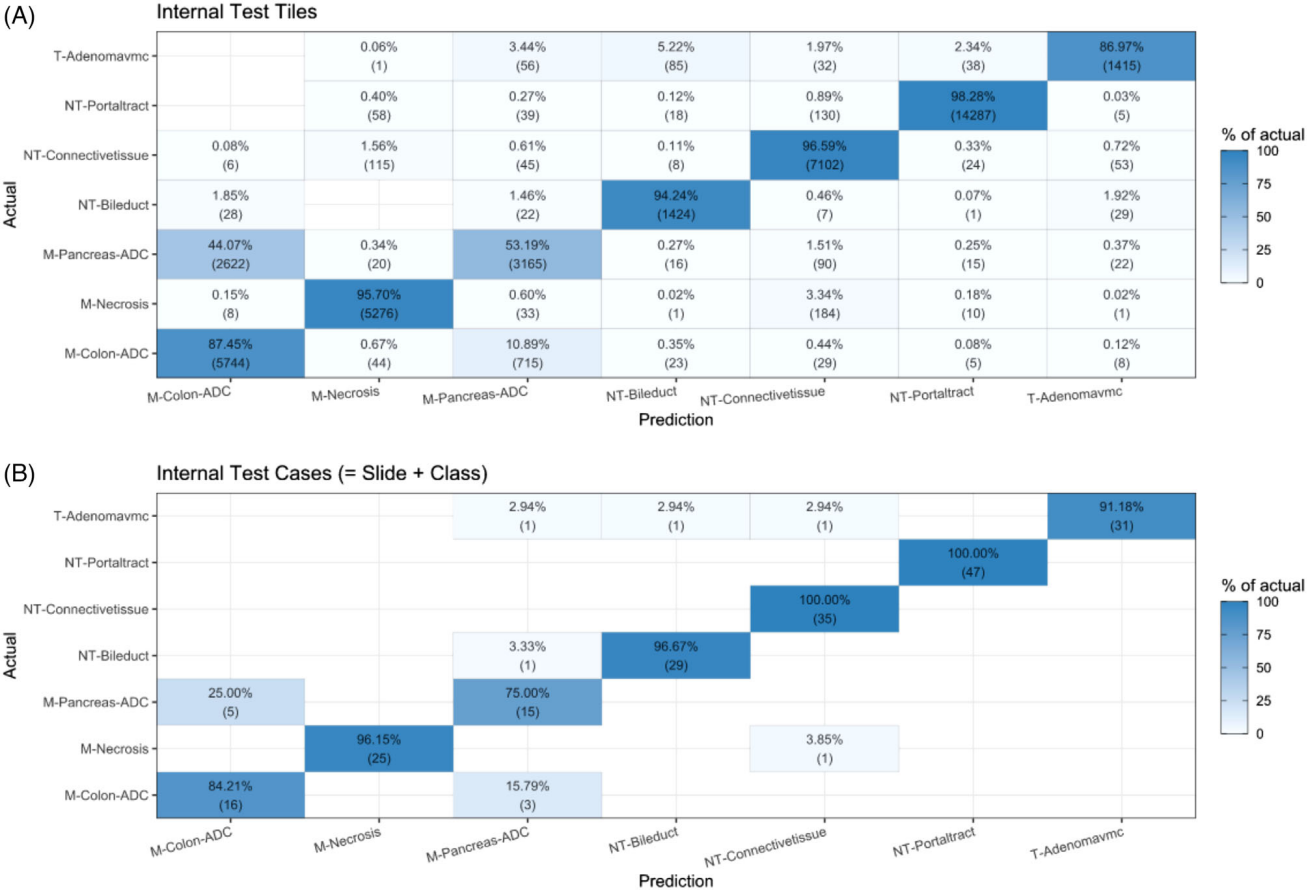
Test data confusion matrices based on image tiles (A) and cases (B). On the level of image tiles and cases, the main misclassification was cases of pancreatic adenocarcinoma predicted as colorectal adenocarcinoma.

On the level of image tiles, common misclassifications included pancreatic adenocarcinoma predicted as colorectal adenocarcinoma (44%) or vice versa (11%). It was uncommon that benign anatomical tissue structures or benign lesions were predicted as malignant tumours. The most common misclassification in this regard occurred with benign bile duct lesions, which were misclassified as colorectal adenocarcinoma (2%). It was also not common that malignant tumours were misclassified as normal hepatic cells or portal tract structures (2%). At the case level, where all tiles of a specific class form one slide/patient, adenocarcinoma metastasis (colorectal and pancreatic) where never misclassified as benign tissue, when using majority vote for the case predicition. Two cases of anatomical tissue structures or benign lesions were misclassified as malignant (colorectal or pancreatic adenocarcinoma). Five cases of pancreatic adenocarcinoma (3%, 5/188) were misclassified as colorectal adenocarcinoma. The respective confusion matrices based on image tiles and cases are shown in Figure 5A,B. Importantly, malignant cases were not misclassified as benign. Together, the low rates reflect the high diagnostic accuracy of our model.

### 3.3 Limitations

**FIGURE 6:**
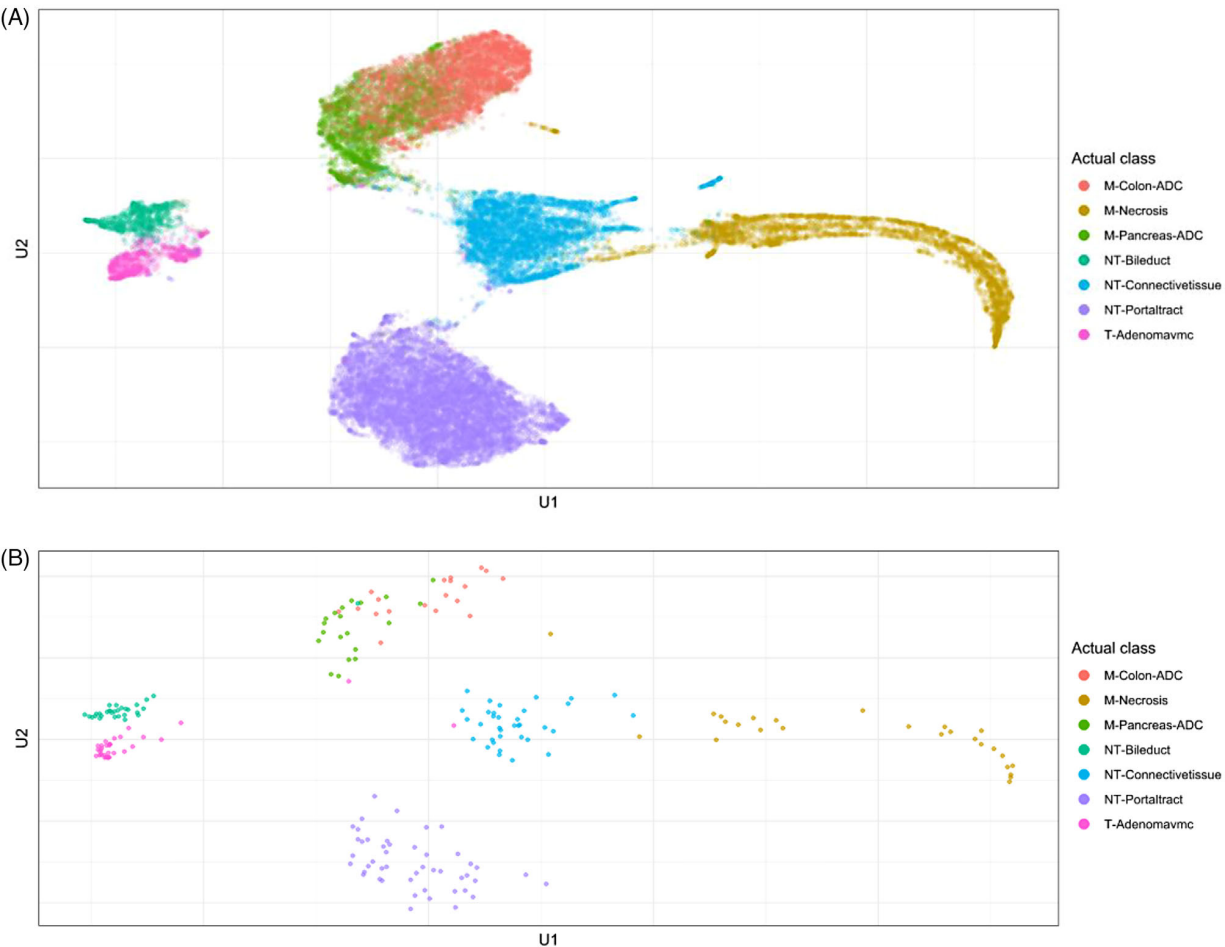
Uniform manifold approximation and projection of the test set based on tiles (A) and patients (B). Dimensionality reduction allows to recognize classes that are similar for the algorithm. Similar classes for the algorithm show close proximity, while classes that are not similar are displayed with a larger distance.

While the confusion matrices of our final model (Figure 5) and the UMAP of the activation layer (Figure 6) show a highly satisfactory separation between classes overall, they also indicate that the discrimination between pancreatic adenocarcinoma and colorectal adenocarcinoma remain difficult for the model: 25% of our pancreatic adenocarcinoma cases were misclassified as colorectal adenocarcinoma and ∼15% of the colorectal adenocarcinoma cases as pancreatic adenocarcinoma. In addition, the UMAP shows a very large overlap with these two classes. For routine diagnostics, these respective classifications may require additional validation. That the data used for training, validation, and testing are relatively homogeneous (based on the preparing pathological institute and the used scanner), may also influence the performance of our models on other data. Nonetheless our study shows that discrimination of the classes in our data seems plausible and our published data and code allow to improve on the limitations.

## 4 DISCUSSION

Digital pathology, where images are reviewed on a computer after high‐resolution scanning of tissue on glass slides, is an emerging method used routinely in the clinical diagnostic setting.^23^ In the present study, we describe the digital assessment of histopathological slides, which has several potential advantages compared to the analog microscopy. First, pathologists can work remote, which allows rapid consultation of cases where special expertise is needed, but not locally available.^23^ Moreover, in emergencies, proven important during the coronavirus pandemic 2019/2020, this method could ensure full availability of pathological diagnostics,^24^ and finally, adapted clinical working times, which may raise surgical pathology to a more personal life situation compatible specialization in medicine. The use of digital pathology tools will improve the overall attractiveness to the field of surgical pathology, which is required to sustain the increase in numbers of complex cases and decline in specialized pathologists.^7^ Second, digital scanning has the advantage of automatic quality control, which can be performed for each slide, for example, to test for equivalency of block and slide numbers or to assess correct staining. Thus, some steps in the workflow, such as assigning slides to cases or archiving, can be accelerated. Third, digital images are usually saved on local hard drives for a certain period of time, which allows a rapid comparison of current to prior specimen. Besides the aforementioned infrastructural features, digitalization of tissue sections allows the application of deep learning algorithms with the potential to support the objective, consistent clinical and diagnostic decision making.^25^, ^26^

Deep learning algorithms and CNN have been previously used to classify benign and malignant diseases on conventionally stained, scanned histopathological slides in various organs such as skin,^12^, ^27^ lung,^10^, ^13^ breast,^28^, ^29^ prostate,^30^, ^31^ or intestinal tissue.^11^, ^32^ In addition, it was used for the assignment of the tumour origin in unknown primary cancers using a metastasized tumours from different anatomical sites (e.g., lymph node, liver), including different types of adenocarcinoma, squamous cell carcinoma, renal, or urothelial carcinoma; although without discrimination of adjacent non‐neoplastic or benign similarly appearing structures,^33^ as shown here.

For hepatobiliary and pancreatic tumours, there are considerably much less publications on deep learning histopathological classification algorithms.^14^, ^34^, ^35^, ^36^ Importantly, deep learning classification to differentiate histologically similar appearing benign structures within the liver, such as non‐neoplastic biliary ducts or benign biliary tumours, from the far most common malignant liver tumours, namely metastasis of colorectal and pancreatic adenocarcinoma, has not been investigated yet. This might be due to the fact that besides liver biopsies, liver surgery is routinely performed only in highly specialized cancer centers. Moreover, it is uncommon that resection of liver metastases of pancreatic adenocarcinoma is performed, since prior studies suggested no survival benefit of surgery in this setting.^37^ Therefore, large numbers of the respective tissue samples are lacking. In our study, we included a series of 103 cases for liver metastases of colorectal adenocarcinoma and notably, 101 of pancreatic adenocarcinoma. In particular, the latter is a considerably large cohort given the above‐mentioned restrictions. Summarizing all classes, the overall number of patients and importantly image patches (204.159) used in this study is far above the range reported in other studies on gastrointestinal and hepatobiliary tumours.^32^

The pipeline used in this study is based on the EfficientNetV2 and the ResNetRS50 architectures. The EfficientNetV2 architecture is a refinement from its initial version, which saves computational resources and outperforms many common deep learning architectures on histopathological tasks.^21^, ^38^ The architecture has been applied in previous studies and achieved high image classification accuracies on the ImageNet reference dataset.^39^ In order to find a well performing model, we have applied common techniques, such as image augmentation.^21^ Although there is currently no gold standard, how to split the dataset into training, validation, and test set, a proportion of 60% ‐ 20% ‐ 20% has been performed previously.^40^

Most studies on the classification of benign and malignant tumours on histopathological tissue sections focus on the differentiation of only few different classes, usually tumour and non‐tumour, or two different tumour classes.^34^, ^41^ Although there are few exceptions,^42^ the vast majority of deep learning studies do not provide annotated data, a full dataset of image patches and/or codes, resulting in published data and algorithms that cannot be independently validated or improved. To address these two issues, we have annotated seven categories in liver tissue including normal anatomical tissue structures, benign and malignant lesions and provided the dataset and code. Our data will allow clinical pathologists and researchers to validate their results and develop novel deep learning methods to support histopathological diagnostics and ultimately, to implement these tools in patient care clinics. By providing these data from normal anatomical tissue structures, our algorithm can in principle be applied to classify whole slide sections, which avoids manual and time consuming annotation of tumour regions prior to classification (see Figure 1).

The performance of our model is highly satisfactory and especially within the reported range, or even outperforming it, when comparing to other deep learning algorithms on histopathological images as reported in other studies.^10^, ^12^, ^32^ It is remarkable that the model performed very well on the differentiation of benign bile duct lesions such as peribiliary hamartoma and biliary adenoma from metastasis from pancreatic adenocarcinoma, a particularly challenging task even for specialized and experienced pathologist. The most common misclassification on the image tile level occurred for pancreatic predicted as colorectal adenocarcinoma (44%). On the case level, five out of a total of 20 cases were misclassified for this task (25%). For further optimization, a larger number of patients would be necessary, a limitation of our study. However, since the decision making of adenocarcinoma was correct with very high confidence in all cases, the final histological differentiation of colorectal and pancreatic adenocarcinoma could be validated by further immunohistological stainings. In this regard, we believe that our algorithm shows a considerably strong and applicable performance, since final decision is still made by surgical pathologists.

It is important to note that our algorithm has been trained only on two different malignant tumour entities. Thus, other tumours not included in the training set cannot be recognized by our model. As a result, our deep learning model can be used as a supplementary diagnostic tool and should always be validated by an expert pathologist to avoid misinterpretations.

In summary, we show for the first time that a comprehensive series of automated identification and classification of common benign and malignant lesions in the liver is possible by deep learning on scanned histological tissue sections. Our work can contribute to an objective and efficient workflow in routine diagnostics for highly relevant diagnostic questions, such as the differentiation between benign and malignant structures and the origin of frequent types of metastasis. This tool may aid pathologists, especially in situations where limited tissue is available, to establish and confirm the diagnosis. Furthermore, we provide an exceptional annotated liver dataset for the development and validation of deep learning algorithms which we provided to the scientific community. At the end, this may be a step towards improved personalized oncology therapy concepts, which will in the future integrate large clinical, radiological and pathological data sets using artificial intelligence.^43^

## CONFLICT OF INTEREST STATEMENT

The authors declare no conflict of interest.

## Supporting information

Supplemental Information
