# Supplementary material for: Implementation of deep learning in liver pathology optimizes diagnosis of benign lesions and adenocarcinoma metastasis
Source: Clin Transl Med. 2023 Jul 6;13(7):e1299. doi: 10.1002/ctm2.1299 (PMC10326372; doi:10.1002/ctm2.1299)
Supplement: Supplementary file 1 — Supplemental Information [file CTM2-13-e1299-s001.docx]

**Supplemental informations and legends**

A


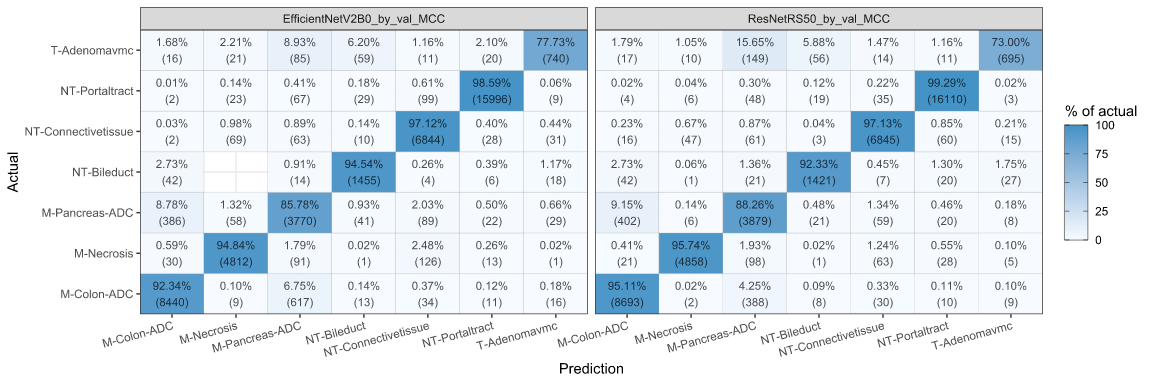


B


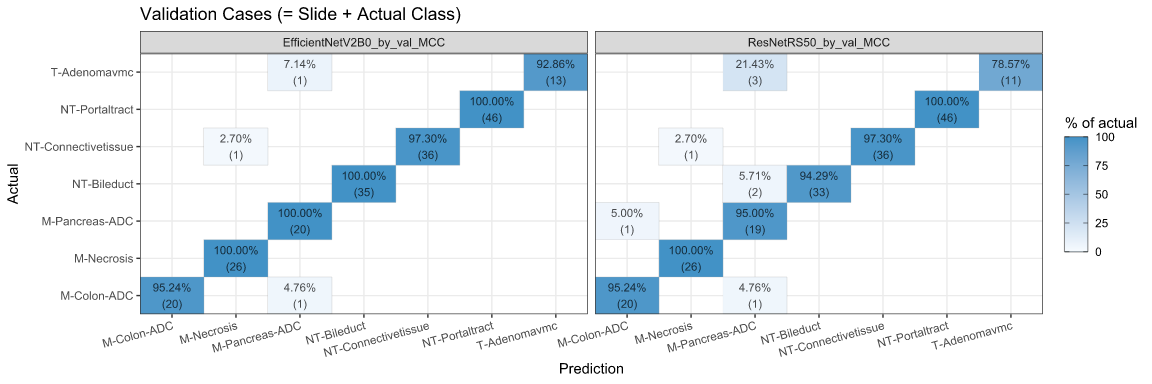


**Suppl. Figure 1: Validation data confusion matrices based on image tiles (A) and cases (B).** On the level of image tiles, there was a slight advantage of the EfficientNetV2B0 model as compared to the ResNetRS50 model, which was also observed on the case level.

A


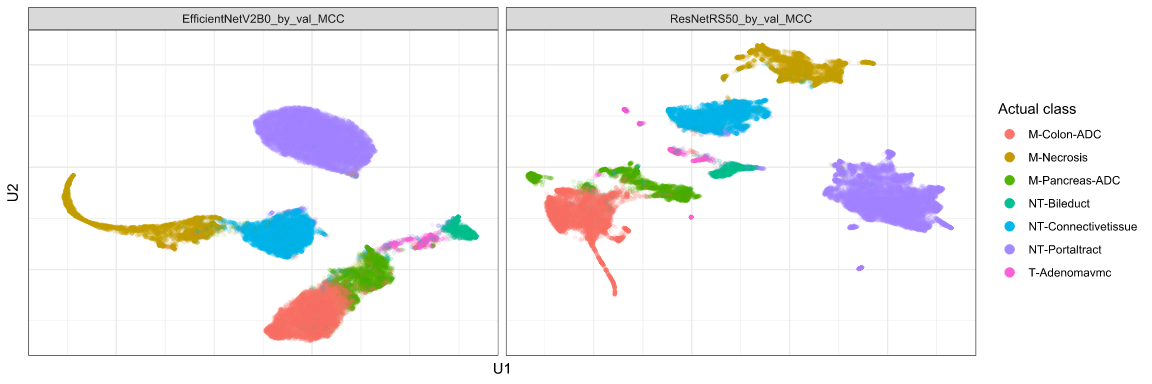


B


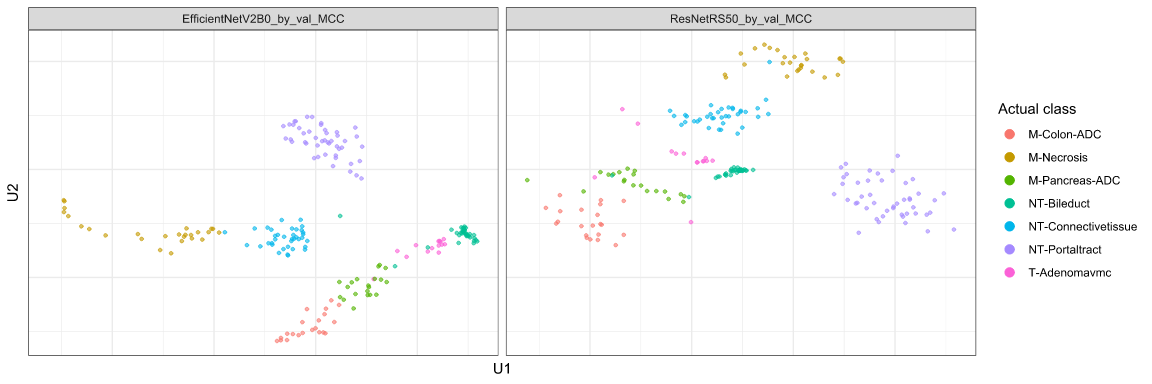


**Suppl. Figure 2: Uniform manifold approximation and projection of the validation set based on tiles (A) and cases (B).** Dimensionality reduction allows recognition of classes that are similar for the algorithm. Similar classes for the algorithm show close proximity, while classes that are not similar are displayed with a larger distance.
